# Neurocysticercosis Prevalence and Characteristics in Communities of Sinda District in Zambia: A Cross-Sectional Study

**DOI:** 10.1007/s44197-024-00271-z

**Published:** 2024-07-09

**Authors:** Gideon Zulu, Dominik Stelzle, Sarah Gabriël, Chiara Trevisan, Inge Van Damme, Chishimba Mubanga, Veronika Schmidt, Bernard J. Ngowi, Tamara M. Welte, Pascal Magnussen, Charlotte Ruether, Agnes Fleury, Pierre Dorny, Emmanuel Bottieau, Isaac K. Phiri, Kabemba E. Mwape, Andrea S. Winkler, the SOLID consortium

**Affiliations:** 1 Ministry of Health, Lusaka, Zambia; 2 Department of Clinical Studies, School of Veterinary Medicine, University of Zambia, Lusaka, Zambia; 3 Department of Neurology, School of Medicine and Health, Technical University of Munich, Munich, Germany; 4 Centre for Global Health, School of Medicine and Health, Technical University of Munich, Munich, Germany; 5 Department of Translational Physiology, Infectiology and Public Health, Faculty of Veterinary Medicine, Ghent University, Ghent, Belgium; 6 Department of Public Health, Institute of Tropical Medicine, Antwerp, Belgium; 7 National Institute for Medical Research, Muhimbili Medical Research Centre, Dar es Salaam, Tanzania; 8 University of Dar es Salaam, Mbeya College of Health and Allied Sciences, Mbeya, Tanzania; 9 Department of Neurology, Epilepsy Center, University Hospital Erlangen, Erlangen, Germany; 10 Department of Immunology and Microbiology, Faculty of Health and Medical Sciences, University of Copenhagen, Copenhagen, Denmark; 11 Department of Radiology, Faculty of Medicine, Technical University of Munich, Munich, Germany; 12 Instituto de Investigaciones Biomédicas-UNAM/Instituto Nacional de Neurología y Neurocirugía/Facultad de Medicina-UNAM, Mexico City, Mexico; 13 Department of Biomedical Sciences, Institute of Tropical Medicine, Antwerp, Belgium; 14 Department of Clinical Sciences, Institute of Tropical Medicine, Antwerp, Belgium; 15 Department of Community Medicine and Global Health, Institute of Health and Society, Faculty of Medicine, University of Oslo, Oslo, Norway; 16 Department of Global Health and Social Medicine, Harvard Medical School, Boston, MA USA

**Keywords:** *Taenia solium*, Cysticercosis, Neurocysticercosis, Point-of-care test, Zambia, Prevalence

## Abstract

**Background:**

This study aimed at describing the epidemiology of (neuro)cysticercosis as well as its clinical and radiological characteristics in a *Taenia solium* endemic district of Zambia.

**Methods:**

This was part of a cross-sectional community-based study conducted in Sinda district to evaluate an antibody-detecting *T. solium* point-of-care (TS POC) test for taeniosis and (neuro)cysticercosis. All TS POC cysticercosis positive (CC+) participants and a subset of the TS POC cysticercosis negative (CC-) received a clinical evaluation and cerebral computed tomography (CT) examination for neurocysticercosis (NCC) diagnosis and staging.

**Results:**

Of the 1249 participants with a valid TS POC test result, 177 (14%) were TS POC CC+ . Cysticercosis sero-prevalence was estimated to be 20.1% (95% confidence intervals [CI] 14.6–27.0%). In total, 233 participants received a CT examination (151 TS POC CC+ , 82 TS POC CC-). Typical NCC lesions were present in 35/151 (23%) TS POC CC+ , and in 10/82 (12%) TS POC CC- participants. NCC prevalence was 13.5% (95% CI 8.4–21.1%) in the study population and 38.0% (95% CI 5.2–87.4%) among people reporting epileptic seizures. Participants with NCC were more likely to experience epileptic seizures (OR = 3.98, 95% CI 1.34–11.78, *p* = 0.01) than those without NCC, although only 7/45 (16%) people with NCC ever experienced epileptic seizures. The number of lesions did not differ by TS POC CC status (median: 3 [IQR 1–6] versus 2.5 [IQR 1–5.3], *p* = 0.64). Eight (23%) of the 35 TS POC CC+ participants with NCC had active stage lesions; in contrast none of the TS POC CC- participants was diagnosed with active NCC.

**Conclusion:**

NCC is common in communities in the Eastern province of Zambia, but a large proportion of people remain asymptomatic.

**Supplementary Information:**

The online version contains supplementary material available at 10.1007/s44197-024-00271-z.

## Introduction

Neurocysticercosis (NCC) is the most common parasitic disease of the central nervous system globally [1]. The disease may occur after accidental ingestion of *Taenia solium* eggs excreted by persons infected with an intestinal *T. solium* tapeworm (taeniosis), which is acquired by consumption of undercooked pork containing *T. solium* cysticerci. NCC is common in low-income and middle-income countries (LMICs) and primarily related to poor sanitation and hygiene, free-range pig husbandry, and improper slaughterhouse practices [2, 3]. Globalization and immigration, however, have led to NCC being also increasingly reported outside LMICs [4, 5]. NCC accounts for about 30% of all acquired epilepsy cases in endemic areas [6]. It presents as two main forms, parenchymal and extra-parenchymal. The clinical presentation of patients is, amongst other factors, primarily determined by cyst localization and the inflammatory response [7, 8]. Cysts within the brain parenchyma or in the cortical sulci in the subarachnoid space often cause epileptic seizures that generally respond well to anti-seizure medication [9]. In contrast, extra-parenchymal NCC in the ventricles or basal cisterns often has a severe course with poor outcome, requiring prolonged treatment with high rates of mortality and disability [8, 10].

Several studies have assessed the high burden and economic impact of porcine and human cysticercosis (CC) in different parts of Zambia [11, 12]. Studies carried out in pigs in the Eastern, Southern and Western provinces indicated high prevalence proportions of porcine cysticercosis, ranging from 15 to 34% based on detection of circulating antigen (Ag-ELISA) [13, 14]. Based on full carcass dissection, prevalence proportions of 46% and 68% were detected in slaughter age pigs in two districts of the Eastern province [15].

Endemicity of *T. solium* infections in humans was confirmed in different studies in the Eastern province of Zambia with taeniosis prevalence proportions ranging from 6.3 to 12% based on copro-Ag-ELISA, and with cysticercosis sero-prevalence ranging from 5.8% to 13% (based on serum Ag-ELISA) and from 34 to 39% (based on serum antibody detection) [2, 12, 16, 17]. Prevalence of NCC in people with epilepsy was also estimated to be over 50% within the same province [18].

Whilst data on human cysticercosis sero-prevalence are available, data on epidemiology and clinical presentation of NCC are largely lacking in sub-Saharan Africa [19], as neuroimaging, the cornerstone of NCC diagnosis, is virtually non-available in rural Africa, including Zambia. In addition, with the wide variation of radiological features for NCC between regions, there is very little information from within Africa, although such data are essential to accurately estimate the burden of NCC in different communities [12, 20–22].

This study aimed to calculate the CC sero-prevalence and NCC prevalence in Sinda district of the Eastern province of Zambia, to compare the clinical features of people with and without NCC, and to describe neuroradiological features of people with NCC. Additionally, we aimed to assess the prevalence of NCC among people with epileptic seizures (PWE) and to describe the differences in clinical and neuroradiological characteristics between PWE with NCC and those without NCC.

## Methods

### Study Design

This study was part of the SOLID project “Evaluation of an antibody detecting point-of-care test for the diagnosis of *Taenia solium* taeniosis and (neuro)cysticercosis in Zambia”, of which detailed procedures, and objectives are published elsewhere [23]. In short, the project aimed at evaluating a novel *T. solium* point-of-care (TS POC) antibody-detecting prototype test for the diagnosis of *T. solium* taeniosis (T), cysticercosis (CC) and NCC. The test is an in-house produced standard lateral flow assay (LFA) based on two recombinant proteins, rES33 and rT24H [24]. This TS POC test was also assessed in patients with neurological signs/symptoms compatible with NCC (epileptic seizures and/or severe progressive headache) in hospital settings in Tanzania, where it was found to have a sensitivity and specificity of 49% (95% CI 41–58%) and 91% (95% CI 89–94%), respectively, for diagnosis of NCC [25]. In the main study in Zambia in which this one is embedded, the sensitivity was estimated to be 26% (95% CI 14–44%) and the specificity 88% (95% CI 85–90%) for NCC diagnosis [26].

**Fig. 1:**
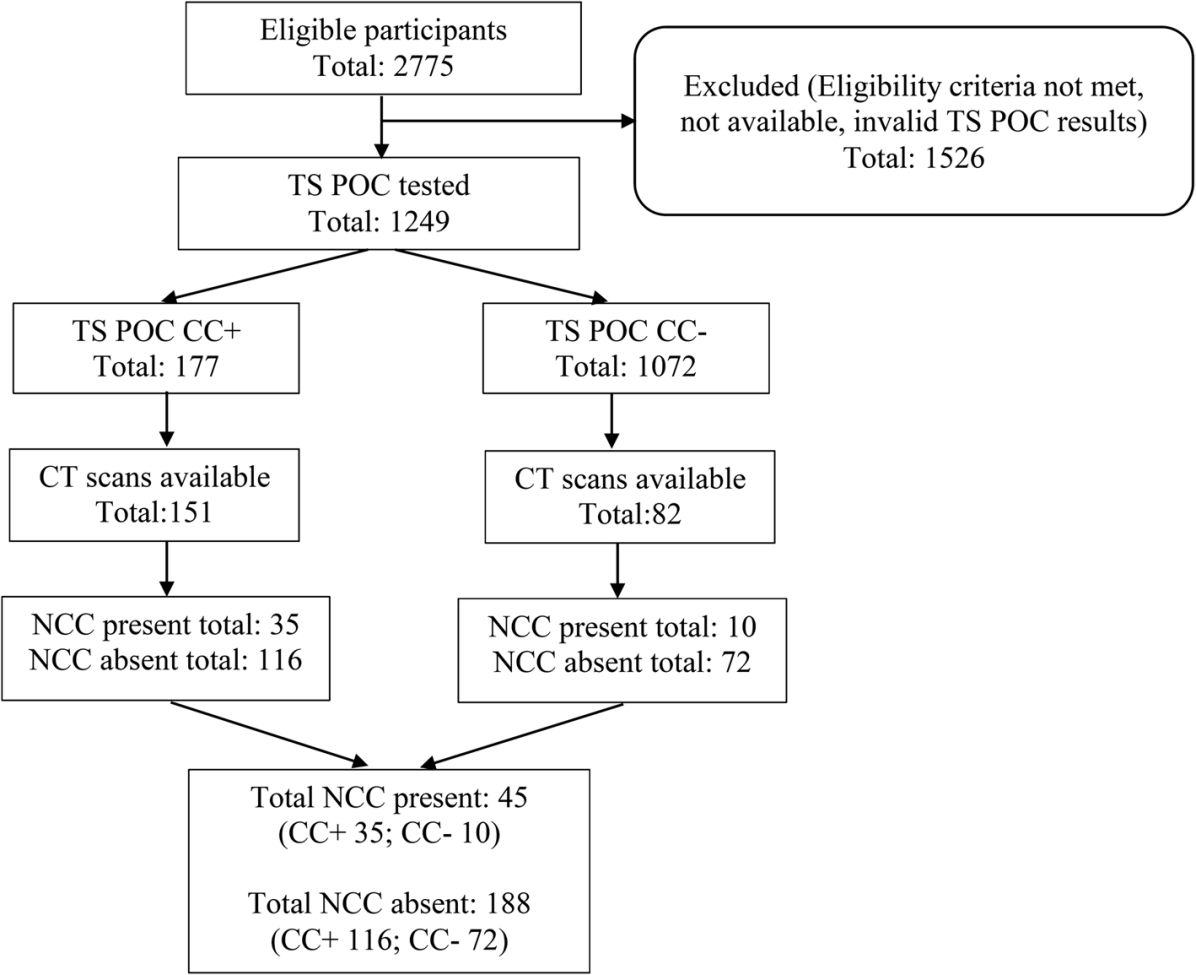
Flowchart for selection of participants for CT examination based on the TS POC CC test result

The study was conducted between December 2017 and November 2021 in Sinda district, Eastern province of Zambia and followed a cross-sectional and community-based design [24]. In short, four (Butao, Chinzure, Mtore, and Ndaula) Neighbourhood Health Communities (NHC; hereafter named “communities”) comprising 40 villages were included. Within each village, households were randomly selected from a list of households established in a census one year prior to the study, resulting in a study population of 2775 people, who were visited to offer TS POC testing. The inclusion criteria were living in the area and being at least 10 years old. Those who were pregnant or reported to be severely ill were excluded from the study. All eligible consenting household members were included in the study and were tested with the TS POC test. Every participant who tested positive for CC on the TS POC test (i.e. TS POC T + CC+ or TS POC T-CC+) and every tenth who tested negative for CC (i.e. TS POC T + CC- or TS POC T-CC-) was requested to give a blood sample and invited for a clinical work-up and cerebral computed tomography (CT) examination (Fig. 1). The blood sample was used for serological testing for CC using the rT24-EITB and the B158/B60 Ag-ELISA as reference tests.

### Clinical Characteristics

The clinical work-up of the selected participants comprised an in-depth medical history, a general physical and neurological examination as well as CT examination with and without contrast. The medical history included collecting demographic data as well as information regarding seizure history and semiology. Everyone coming for clinical work-up was screened for epileptic seizures using a validated questionnaire (A1 Table) [21]. Headache assessment started with the screening question whether people regularly experienced headache; among all people who regularly experience headache, information on location, intensity, duration, frequency, and quality of the headache was also collected. Headache intensity was assessed using a pictorial headache intensity score chart. The neurological examination involved assessment of the patients’ cranial nerves, muscle strength, muscle tone, reflexes, extrapyramidal system, coordination and gait, sensory system, and mental state.

### Radiological Assessment

CT examinations were performed at the Chipata central hospital. We used the Neusoft helical multi-slice scanner (Neusoft Medical Systems, Shenyang, China), which is a 16-slice machine with a slice thickness of 1.5 mm. The evaluation of CT scans and diagnosis of NCC followed the same procedure as described by Stelzle et al., 2022 [19]. In short, CT scans were evaluated by two independent reviewers (CR: neuroradiologist; AF: NCC specialist and neurologist) who were blinded to the TS POC test results. A third reviewer (ASW: neurologist) adjudicated in case of disagreement. Diagnosis of NCC was made following the principles of the revised Del Brutto criteria [27] and the stage of NCC lesions was categorized as active (viable cysts: vesicular stage; degenerating cysts: colloidal or granular nodular stage) or inactive (calcified stage) [28, 29]. Depending on their location the lesions were described as parenchymal (frontal lobe, temporal lobe, parietal lobe, occipital lobe, cerebellum, brainstem) or extra-parenchymal (intraventricular, subarachnoid in basal cisterns, and subarachnoid in cortical sulci). The radiological as well as the clinical assessment followed the same procedures as the SOLID project in Tanzania [19, 25].

### Statistical Analyses

The first part of this study was an epidemiological assessment of CC and NCC prevalence in the study district. Cysticercosis sero-prevalence was calculated based on the cysticercosis reference test results taking into account the study design (i.e. the TS POC CC result). The reference tests were the rT24H-EITB and the B158/B60 Ag-ELISA. Details on the test procedures have been published elsewhere [24]. All prevalence proportions were determined using survey-weighted generalized linear models [30, 31]. Weights were determined using the two-phase function [31] including the TS POC test result combination as stratum identifier in phase two and including households as cluster variable. This approach is used to account for the two-phase design and assumes that the reference standard is missing at random given the TS POC test result combination of both test strips.

For the second part of the study, total number and proportions were used to describe the demographical, clinical, and radiological characteristics of NCC, disaggregated by TS POC CC result. Differences in these factors between people with and without NCC were compared using logistic regression models adjusted for the TS POC CC result. Differences in the number of NCC lesions by TS POC CC result were analysed using Wilcoxon rank sum tests. Clinical characteristics between PWE with and without NCC were only described and no statistical test was performed because of the small numbers. For categorical variables, numbers and proportions were presented, whereas the median and interquartile range (IQR) were presented for continuous variables. A p-value of < 0.05 was considered as statistically significant. R version 4.1.1 was used to perform all analyses.

## Results

### Prevalence of Cysticercosis and Neurocysticercosis

Across the four communities, a total number of 1249 of participants had a valid TS POC test result with 224 (18%) from Butao, 293 (23%) from Chinzure, 438 (35%) from Mtore, and 294 (24%) from Ndaula (Table 1). The median age for participants was 28 years (interquartile range 18 to 44 years). Twenty percent were children under the age of 15 years. More females than males were recruited (57% versus 43%). Of all people included, 1072 (86%) participants were negative for CC on the TS POC test, and 177 (14%) participants were CC+ with variation across communities (p < 0.001). In all villages at least one person was CC+ apart from two villages in which only five and eight people were included. Applying these results with the results of the reference tests, as published previously by Mubanga et al.[24], yielded a cysticercosis sero-prevalence of 20.1% (95% CI 14.6–27.0%) for at least one of the two cysticercosis reference tests being positive; Cysticercosis sero-prevalence based on antigen ELISA alone was 13.3% (95% CI 8.9–19.5%) and 8.8% (95% CI 5.4–13.9%) based on the rT24H-EITB (Table 2).

**Table 1:** Demographic characteristics and TS POC test results of study participants

	Overall	Community				p-value
		Butao	Chinzure	Mtore	Ndaula	
N	1249	224	293	438	294	
Age group						
(10,20]	409 (33%)	76 (34%)	98 (33%)	141 (32%)	94 (32%)	0.49
(20,40]	466 (37%)	82 (37%)	99 (34%)	172 (39%)	113 (38%)	
(40,60]	256 (20%)	43 (19%)	63 (22%)	82 (19%)	68 (23%)	
(60,80]	98 (8%)	20 (9%)	29 (10%)	32 (7%)	17 (6%)	
(80,100]	20 (2%)	3 (1%)	4 (1%)	11 (3%)	2 (1%)	
Age in years						
Median [IQR]	28 [18, 44]	29 [17, 43.3]	27 [17, 45]	27 [18, 44]	30 [18, 43]	0.97
Sex						
Female	714 (57%)	126 (56%)	174 (59%)	235 (54%)	179 (61%)	0.21
Male	535 (43%)	98 (44%)	119 (41%)	203 (46%)	115 (39%)	
Households						
Number	506	91	124	189	102	
Mean number of people per household	2.5	2.5	2.4	2.3	2.9	
TS POC test result						
T– CC-	1066 (85%)	195 (87%)	221 (75%)	393 (90%)	257 (87%)	< 0.001
T– CC+	170 (14%)	25 (11%)	69 (24%)	42 (10%)	34 (12%)	
T + CC-	6 (0%)	3 (1%)	2 (1%)	0 (0%)	1 (0%)	
T + CC+	7 (1%)	1 (0%)	1 (0%)	3 (1%)	2 (1%)	
TS POC CC						
CC–	1072 (86%)	198 (88%)	223 (76%)	393 (90%)	258 (88%)	< 0.001
CC+	177 (14%)	26 (12%)	70 (24%)	45 (10%)	36 (12%)	
Village level prevalence						
Median [IQR]	13% [8–20%]	12% [10–13%]	24% [17–27%]	11% [7–18%]	13 [12–20%]	
Household with at least one TS POC CC+ person	140 (28%)	24 (26%)	48 (39%)	38 (20%)	30 (29%)	

*TS POC* *Taenia solium* point-of-care, *CC+* cysticercosis positive, *CC–* cysticercosis negative

**Table 2:** Prevalence proportions of cysticercosis, (active) neurocysticercosis, epileptic seizures and neurocysticercosis among people with and without epileptic seizures

	TS POC CC+	TS POC CC-	Prevalence	[95% CI]
Participants	177	1072		
CC serology				
At least one reference test positive	43/138 (31%)	22/120 (18%)	20.1%	[14.6–27.0%]
Both reference tests positive	13/138 (9%)	1/120 (1%)	2.1%	[0.9–4.5%]
Antigen ELISA positive	29/140 (21%)	14/126 (11%)	13.3%	[8.9–19.5%]
rT24H-EITB positive	27/138 (20%)	9/120 (8%)	8.8%	[5.4–13.9%]
NCC				
NCC	35/151 (23%)	10/82 (12%)	13.5%	[8.4–21.1%]
Active NCC	8/151 (5%)	0/82 (0%)	1.1%	[0.6–2.0%]
Epileptic seizures and NCC				
Epileptic seizures	11/151 (7%)	4/80 (5%)	4.1%	[1.8–8.9%]
NCC among people with epileptic seizures	6/11 (55%)	1/4 (25%)	38.0%	[5.2–87.4%]
NCC among people without epileptic seizures	29/140 (21%)	9/76 (12%)	12.6%	[7.5–40.3%]

+  positive, – negative, *CC* cysticercosis, *EITB* enzyme linked immunoelectrotransfer blot, *ELISA* enzyme linked immunosorbent assay, *NCC* neurocysticercosis, *rT24H* recombinant cysticercus protein

Of the 177 TS POC CC+ participants, 151 (85%) had a CT examination (Fig. 1) and of those 35 had NCC (23%), among which eight (5%) were in the active stage. Of the 1072 TS POC CC- participants, 82 had a CT examination and of those, 10 had NCC (12%) with no lesion in active stage. Participants who did not receive a CT examination did not differ demographically from those with a CT examination (A2 Table). Accounting for the study design, a prevalence of 13.5% (95% CI 8.4–21.1%) for any type of NCC and 1.1% (95% CI 0.6–2.0%) for active stage NCC was estimated. The estimated lifetime prevalence of epileptic seizures in the study population was 4.1% (95% CI 1.8–8.9%) and the NCC prevalence among PWE was 38.0% (95% CI 5.2–87.4%) (Table 2).

### Characteristics of Participants with Neurocysticercosis

Overall, 233 participants underwent CT examination. Of those, 45 (19%) had NCC-typical lesions. Males and females were equally affected (OR = 1.68, 95% CI 0.86–3.39, p = 0.13). Participants with NCC were on average 5.9 years (95% CI – 0.4 to 12.2 years) older than those without NCC (p = 0.07, Table 3). One of the 27 (4%) children (between 10 and 14 years) who received a CT examination, had NCC. Small-scale farmers more commonly had NCC than participants of other occupations (OR = 5.28, 95% CI 1.56–17.86, p = 0.007). Sero-positivity in the reference tests was statistically significantly associated with NCC, but nonetheless more than 10% of people with negative serology had NCC. Overall, 15/231 participants irrespective of the TS POC test result (6%) screened positive for epileptic seizures, and participants who reported epileptic seizures more commonly had NCC than those who did not report epileptic seizures (OR = 3.98, 95% CI 1.34–11.78, p = 0.01) (Table 3). Yet, there were no marked differences in terms of seizure semiology, frequency, or anti-seizure medication (A3 Table). Of all participants with NCC, 16% (7/45) had ever experienced an epileptic seizure in their life. Among those with active NCC, 36% (4/11) had ever experienced an epileptic seizure in their life while only 9% (3/34) among those with inactive NCC, experienced an epileptic seizure. Thirty-three percent (15/45) of participants with NCC reported experiencing headaches regularly and this was of varying intensities, quality, duration and frequency (Table 3, A4 Table). Overall, among the participants who reported experiencing headaches regularly, there were more without NCC compared to those with NCC, though the difference was not significant (OR = 1.74, 95% CI 0.85–3.59, p = 0.13; Table 3). The majority of the participants with NCC reportedly neither experienced an epileptic seizure (84%; 38/45) nor regular headaches (67%; 30/45).

**Table 3:** Demographic and relevant clinical description of people with and without neurocysticercosis by TS POC CC result

	Level	Overall	TS POC CC positive		TS POC CC negative		Odds Ratio (95% CI)	p-value^£^
			NCC n *(%)	No NCC n *(%)	NCC n *(%)	No NCC n*(%)		
n		233	35 (23)	116 (77)	10 (13)	72 (88)		
Sex	Male	120	22 (28)	57 (72)	6 (15)	35 (85)	1.68 (0.86–3.30)	0.13
	Female	113	13 (18)	59 (82)	4 (10)	35 (90)	Reference	
Age in years	Median [IQR]	32 [20, 48]	43 [26, 50]	31 [17, 44]	40 [29, 44]	30 [20, 48]	5.9 years (– 0.4 to 12.2 years)	0.07
Age group in years	(10,20]	62	5 (12)	38 (88)	1 (5)	19 (95)		
	(20,40]	86	12 (23)	41 (77)	4 (12)	29 (88)		
	(40,60]	53	13 (36)	23 (64)	3 (18)	14 (82)		
	(60,80]	26	4 (27)	11 (73)	2 (18)	9 (82)		
	(80,100]	4	1 (25)	3 (75)	0	0		
Religion	Christian	195	65 (90)	7 (10)	91 (74)	32 (26)	0.72 (0.27–1.87)	0.50
	Other or no religion	36	5 (63)	3 (37)	25 (89)	3 (11)	Reference	
Occupation	Small-scale farmer	177	51 (38)	84 (62)	9 (21)	33 (79)	5.28 (1.56–17.86)	0.007
	Other	54	19 (37)	32 (63)	1 (33)	2 (67)	Reference	
Reference tests (n = 189)	Both reference tests positive	13	7 (58)	5 (42)	0 (0)	1 (100)	6.33 (1.88–21.28)	0.003
	Only antigen ELISA positive	24	3 (23)	10 (77)	2 (18)	9 (82)	1.81 (0.59–5.54)	0.30
	Only rT24H-EITB positive	20	7 (54)	6 (46)	1 (14)	6 (86)	4.35 (1.54–12.26)	0.006
	Both reference tests negative	132	13 (15)	73 (85)	5 (11)	41 (89)	Reference	
Screening positive for epileptic seizures	No	216	29 (21)	111 (79)	9 (12)	67 (88)	3.98 (1.34–11.78)	0.01
	Yes	15	6 (55)	5 (45)	1 (25)	3 (75)	Reference	
Headache regularly	No	177	22 (20)	88 (80)	8 (12)	59 (88)	1.74 (0.85–3.59)	0.13
	Yes	54	13 (32)	28 (68)	2 (15)	11 (85)	Reference	
Neurological deficit	No	232	35 (23)	116 (77)	10 (13)	71 (88)	NA	NA
	Yes	1	0	0	0	1 (100)		

Information missing for two TS POC CC negative participants without NCC

*(%) refers to the number of patients with the reported characteristic

^£^p-value from logistic regression models adjusted for TS POC CC result to account for the study design

From the clinical examination of the 233 participants, only one exhibited a neurological deficit (facial palsy; A3 Table). The rest of the participants with NCC had normal neurological examination findings.

### Radiological Characteristics of Neurocysticercosis

Forty-five participants had NCC, 35 TS POC CC+ and 10 TS POC CC-. Six of the 35 TS POC CC+ participants with NCC (17%) had epileptic seizures, and one of the ten TS POC CC- with NCC (10%). The total number of NCC lesions was 263 for TS POC CC+ participants and 46 for TS POC CC- (Table 4). Median number of lesions did not differ by TS POC CC status (TS POC CC+ : 3 (IQR 1–6) versus TS POC CC-: 2.5 (IQR 1–5.3), p = 0.64). TS POC CC+ participants with epileptic seizures did not have more lesions compared with TS POC CC+ participants who had never experienced epileptic seizures (median: 3 (IQR 2–7) versus 3 (IQR 1–6), p = 0.63). There was also no association between the number of lesions and the likelihood of epileptic seizures (Table A5) though more patients with NCC and epileptic seizures had parenchymal lesions (Table 4). Only the TS POC CC+ participants had active stage lesions – all in vesicular stage. Eight of the eleven (73%) patients with active lesions, also had calcified lesions (i.e. mixed lesions). Individuals with TS POC CC- NCC all had calcified lesions only. All 45 NCC participants had parenchymal lesions, 10 had further extra-parenchymal lesions which were all located in the cortical sulci in the subarachnoid space. There was no association between the likelihood of extra-parenchymal lesions and TS POC CC status.

**Table 4:** Radiological characteristics of people with neurocysticercosis (with and without epileptic seizures) by TS POC CC result

		TS POC CC+ n (%)	TS POC CC+ and epileptic seizures n (%)	TS POC CC+ and no epileptic seizures n (%)	TS POC CC- n (%)^a^	TS POC CC- and epileptic seizures n (%)	TS POC CC- and no epileptic seizures n (%)
Total number of participants with NCC		35	6	29	10	1	9
Participants with mixed (active/calcified) lesions		8/35 (23)	4/6 (67)	4/29 (14)	0/10 (0)	0/1 (0)	0/9 (0)
Participants with only active lesions		3/35 (9)	0/6 (0)	3/29 (10)	0/10 (0)	0/1 (0)	0/9 (0)
Participants with only calcified lesions		24/35 (69)	2/6 (33)	22/29 (76)	10/10 (100)	1/1 (100)	9/9 (100)
Total number of lesions		263	71	192	46	2	44
Median number of lesions per patient [IQR]		3 [1–6]	3 [2–7]	3 [1–6]	2.5 [1–5.3]	NA	3 [1–6]
Total number of active lesions		116 (44)	48 (68)	68 (35)	0 (0)	0 (0)	0 (0)
Total number of calcifications		147 (56)	23 (32)	124 (65)	46 (100)	2 (100)	44 (100)
*Participants with lesions at location*							
Frontal		34 (97)	6 (100)	28 (97)	10 (100)	1 (100)	9 (100)
Temporal		14 (40)	4 (67)	10 (34)	4 (40)	0 (0)	4 (44)
Parietal		9 (26)	0 (0)	9 (31)	0 (0)	0 (0)	0 (0)
Occipital		9 (26)	1 (17)	8 (28)	2 (20)	0 (0)	2 (22)
Brainstem		0 (0)	0 (0)	0 (0)	0 (0)	0 (0)	0 (0)
Cerebellum		2 (6)	2 (33)	0 (0)	1 (10)	0 (0)	1 (11)
Subarachnoid – cortical sulci		8 (23)	3 (50)	5 (17)	2 (20)	0 (0)	2 (22)
Subarachnoid – basal cisterns		0 (0)	0 (0)	0 (0)	0 (0)	0 (0)	0 (0)
Intraventricular		0 (0)	0 (0)	0 (0)	0 (0)	0 (0)	0 (0)
Parenchymal		35 (100)	6 (100)	29 (100)	10 (100)	1 (100)	9 (100)
Extra-parenchymal		8 (23)	3 (50)	5 (17)	2 (20)	0 (0)	2 (22)

*TS POC* *Taenia solium* point-of-care test, *CC+* cysticercosis positive, *CC–* cysticercosis negative

^a^No TS POC CC– participant in the study population had ever experienced an epileptic seizure

## Discussion

In this study we assessed the sero-prevalence of CC and the prevalence of NCC in Sinda district of the Eastern province in Zambia. Although our study used a design based around a TS POC CC test with imperfect accuracy, the modelling of the results made our findings reliable and independent from the accuracy of the TS POC CC test. The TS POC CC test has been evaluated for NCC in a clinical setting, among people with epileptic seizures where it highlighted a limited sensitivity. The sensitivity was, however, comparable to the sensitivity of the current reference tests. Sensitivity of the TS POC CC test was even excellent for the detection of active-stage NCC lesions irrespective of the presence or absence of neurological signs and symptoms [25, 26].

In our study, every seventh participant was cysticercosis positive on the TS POC CC test, but positivity differed considerably between and within health communities with the highest burden being observed in Chinzure and the lowest in Mtore. Cysticercosis prevalence assessed by the current reference tests was even higher with every fifth person of the community having the infection. It is difficult to pinpoint exactly the factors present in the study area or population that can explain the observed higher CC positivity in Chinzure compared to the other three communities.

A sizeable proportion (13.5%) of people had NCC lesions – however, only a small fraction (not even every tenth) of these had lesions in active stage which may indicate a reduction in infection pressure over the last years [32, 33]. Community-based studies of NCC are scarce, but in a study conducted in a *T. solium* endemic area in Peru, 18% of residents had NCC [34].

We also observed that only a small proportion of people with NCC reported having ever had epileptic seizures, and that among people with epileptic seizures, more than every third patient had NCC lesions. As comparison, among people without epileptic seizures, around every eighth person had NCC. This is important, as many epidemiological studies on NCC only include people with epileptic seizures or serologically positive people for further neuroimaging [18, 35]. As a comparison, in a previous study in an adjacent district to our study area, more than 50% of people with epilepsy had NCC [18]. In that study, however, the prevalence of NCC was higher than in our study population probably because only people with active epilepsy were recruited for neuroimaging whereas our study included all people who ever had an epileptic seizure. These findings are consistent with observations from other Latin American countries and globally [6, 36]. The differences between the health communities re-emphasize the varying burden of *T. solium* on a very granular level which has been demonstrated before [20, 37].

We also found that participants with NCC were older than participants without NCC (although not significant) which is consistent with previously published studies [38–40]. Older age not only predisposes to more frequent infection but also to changes in the immune system which over some time protects the host against inflammatory reaction towards the parasite [41].

All NCC patients with epileptic seizures had calcifications on neuroimaging, some had additional active stage lesions. Recurrent seizures are still common in patients with calcified parenchymal brain cysticerci, although their frequency is often lower than in case of active/degenerating stage lesions [42–44]. However, in our study, a good proportion (82%) of participants with calcifications reportedly had never experienced an epileptic seizure. This phenomenon could be explained by the fact that there were relatively few NCC lesions per patient, that epileptic seizures may only occur when lesions are located near the grey matter-white matter junction [45] or by the hypothesis that there may be a genetic predisposition to low seizure threshold in people with NCC who develop epilepsy [46, 47].

Moreover, we found that a substantial proportion of people with NCC, especially those with calcified NCC, were negative in both reference tests. This inaccuracy of the reference tests in detecting calcified lesions has previously been described [25, 40].

Concerning typical neurological signs/symptoms of NCC, we focused on evaluation and analyses of participants with epileptic seizures. Yet, headache is also a typical symptom and the second most encountered manifestation of NCC after seizures [5, 48, 49]. However, although we investigated headache during our clinical assessment, we realized by time of analyses that answers were too inconsistent and unspecific to be evaluated for NCC. Nevertheless, our assessment showed that there is a high burden of headache in our study population (irrespective of NCC status), with about one third of participants reporting regular headache. This is in line with the findings of the Global Burden of Disease study which found a considerable burden of headache that is often not optimally recognized and treated [50]. Headache within the sub-Saharan region are a common finding [51, 52]. An association between calcified NCC and headache was also recently established in a community case–control study conducted in Ecuador [53]. This study also found that the burden of infection had no significant effect on the outcome of the headache among NCC patients. Consequently, for future NCC studies, headache should be evaluated in far greater detail.

## Strengths and Limitations

Our study was community-based and had a relatively large sample size. As we included the TS POC result as a stratification factor for further clinical work-up, we were able to infer the true prevalence of NCC in the total study population, irrespective of inaccuracies of serological tests. Nonetheless, selection bias may have been present as only some households were visited and not everybody living in the households was present during the recruitment and TS POC testing. However, as households were selected at random, we do not think that this has impacted prevalence estimates considerably.

Our study also had some limitations. We are well aware that the serological tests used in our study are not perfect to diagnose cysticercosis [24], but since they are still very often used, the tests were utilized only to allow comparison with previously published studies. In addition, we used a CT scanner and not a magnetic resonance imaging (MRI) for the detection of NCC lesions because MRI is not available in the province. Generally, CT scanners are good for detecting calcified lesions, but may miss some active stage lesions which more likely are captured by MRI. Hence, we may have underestimated the active NCC prevalence; the prevalence of any type of NCC will likely not have been affected considerably. Especially also, because all participants had a CT scan with and without contrast which may have increased likelihood of detection of lesions. For some participants, there was quite a long time between TS POC test and the CT examination. This is because the CT scanner was often dysfunctional which resulted in a postponing of appointments. Furthermore, the CT examinations were done about 120 km away from the study site and participants had to be taken in small groups, which meant they had to spend a whole day to undergo CT examination. These delays may have affected our results. However, in a hospital-based setting among people with epileptic seizures [25], an assessment on the effect of this delay was done and the results were negligible. We thus believe this effect would not be considerably different in our study.

Furthermore, in this study people were asked if they had ever experienced an epileptic seizure. Although a validated questionnaire was used, recall bias and stigma will likely have affected the assessment—however, we do not think that there would be a difference between people with and without NCC.

## Conclusion

NCC is common in Sinda district in Eastern province of Zambia. Active stage lesions, however, are relatively infrequent. We demonstrated that a large proportion of people with NCC reportedly never experienced epileptic seizures and that many people with NCC (especially those with only calcified lesions) are serologically negative for *T. solium* cysticercosis antibodies. The prevalence of NCC in Sinda district communities calls for closer collaboration between human and veterinary public and environmental health services in order to break the *T. solium* lifecycle in humans, pigs and potentially also the environment—in an equitable, interdisciplinary One Health approach with affected communities at its core.

## Supplementary Information

Below is the link to the electronic supplementary material.

Supplementary file1 (DOCX 183 kb)

## Data Availability

All data used are within the manuscript and its supporting information files.
