# Supplementary material for: Neurocysticercosis Prevalence and Characteristics in Communities of Sinda District in Zambia: A Cross-Sectional Study
Source: J Epidemiol Glob Health. 2024 Jul 9;14(3):1180–90. doi: 10.1007/s44197-024-00271-z (PMC11444043; doi:10.1007/s44197-024-00271-z)
Supplement: Supplementary file 1 — Supplementary file1 (DOCX 183 kb) [file 44197_2024_271_MOESM1_ESM.docx]

**Appendix:**

**A1 Table** Screening questionnaire for epileptic seizures

| **N°** | **Question** | **Comment** |
| --- | --- | --- |
| **Screening questionnaire for epileptic seizures** | | |
| 1 | Have you ever been told that you had an episode of losing consciousness during which your arms and legs shake or stretch out? | Yes / No / Do not know |
| 2 | During attacks of unconsciousness have you ever bitten your tongue or lost control of your bladder or bowels? | Yes / No / Do not know |
| 3 | Have you ever had uncontrollable attacks of shaking or trembling in one arm or one leg or in the face without losing consciousness? | Yes / No / Do not know |
| 4 | Have you ever been told that you have or had epilepsy or epileptic seizures? | Yes / No / Do not know |
|  | *Only* ***one Yes*** *is required to screen positive for epileptic seizures.* | Screen positive / Screen negative |

**A2 Table** Differences between participants who underwent computed tomography examination and those who did not, by TS POC CC

|  | **Level** | **TS POC CC positive** | | | **TS POC CC negative** | | |
| --- | --- | --- | --- | --- | --- | --- | --- |
|  |  | **CT performed** | **No CT performed** | **p-value^£^** | **CT performed** | **No CT performed** | **p-value^£^** |
| n | | 151 | 26 |  | 82 | 992 |  |
| n (with demographic information available) | | 151 | 26 |  | 80 | 992 |  |
| Sex | Female | 73 (48) | 14 (54) | 0.76 | 39 (49) | 588 (59) | 0.20 |
|  | Male | 78 (52) | 12 (46) |  | 41 (51) | 404 (41) |  |
| Age in years | Median  [IQR] | 33 [22, 45] | 34 [22, 45] | 0.79 | 29 [19, 45] | 27 [17, 43] | 0.33 |
| Age group in years | (10,20] | 42 (28) | 5 (19) | 0.66 | 19 (24) | 343 (35) | 0.69 |
|  | (20,40] | 54 (36) | 10 (39) |  | 33 (41) | 369 (37) |  |
|  | (40,60] | 37 (25) | 9 (35) |  | 17 (21) | 193 (19) |  |
|  | (60,80] | 14 (9) | 1 (4) |  | 11 (14) | 72 (7) |  |
|  | (80,100] | 4 (3) | 1 (4) |  | 0 (0) | 15 (2) |  |

£ P-value derived from Chi-square test for categorical variables and Wilcoxon rank-sum test for median age in years.; CC, cysticercosis; CT, computed tomography; POC, point-of-care; TS, *Taenia solium.*

**A3 Table** Clinical differences between people with epileptic seizures with and without neurocysticercosis

|  |  | **Epileptic seizures and NCC**  **n (%)** | **Epileptic seizures without NCC**  **n (%)** |
| --- | --- | --- | --- |
| N |  | 7 | 8 |
| TS POC | CC+ | 6 | 5 |
|  | CC- | 1 | 3 |
| Sex | Female | 0 | 4 (50) |
|  | Male | 7 (100) | 4 (50) |
| Age in years | Median [IQR] | 41 [29-48] | 19.5 [16-39.3] |
| Age of seizure onset | Median [IQR] | 7 [1-25] | 2 [1-5.3] |
| Motor activity during seizures | Tonic-clonic | 7 (100) | 7 (88) |
|  | No movement of limbs, but rolling of eyes and grinding of teeth | 0 | 1 (13) |
| Focal onset of seizures |  |  |  |
| Side of onset of seizures | Bilateral | 7 (100) | 7 (88) |
|  | Unilateral | 0 | 0 |
|  | Not applicable | 0 | 1 (13) |
| Loss of consciousness | No loss of consciousness | 0 | 2 (25) |
|  | Yes, after motor signs start | 2 (29) | 0 |
|  | Yes, from the beginning | 5 (71) | 6 (75) |
| Aura |  | 4 (57) | 2 (25) |
| Anti-seizure medication |  | 3 (43) | 2 (25) |
|  | *Single therapy* | *3/3 (100)* | *1/2 (50)* |
|  | *Combination therapy* | *0* | *1/2 (50)* |
| Seizure frequency | Daily to monthly | 0 | 0 |
|  | Monthly to yearly | 3 (43) | 1 (13) |
|  | None any more | 4 (57) | 6 (75) |
|  | Less than yearly, irregularly | 0 | 1 (13) |
| **Risk factors** |  |  |  |
| Illness before first seizure |  | 1 (14) | 1 (13) |
| Family history of epilepsy |  | 3 (43) | 4 (50) |
| **Impact of seizures** |  |  |  |
| Injuries |  | 3 (43) | 4 (50) |
| Schooling |  | 5 (71) | 3 (38) |
| Drop-out from school |  | 3 (43) | 0 |
| **Chronic diseases** |  |  |  |
| Mental health disorders |  | 0 | 1 (13) |
| HIV infection |  | 0 | 0 |
| Neurological examination | Deficit | 0 | 1 (facial paralysis) |

CC+, cysticercosis positive; CC-, cysticercosis negative; IQR, interquartile range; NCC, neurocysticercosis; POC, point-of-care; TS, *Taenia solium.*

**A4 Table** Headache characteristics among people with neurocysticercosis

|  |  |  | **TS POC positive** | | **TS POC negative** | |  |
| --- | --- | --- | --- | --- | --- | --- | --- |
|  | **Level** | **Overall** | **NCC**  **n (%)** | **No NCC**  **n (%)** | **NCC**  **n (%)** | **No NCC**  **n (%)** | **p** |
| n |  | 233 | 35 | 116 | 10 | 72 |  |
| Headache regularly | No | 177 | 22 (20) | 88 (80) | 8 (12) | 59 (88) | 0.11 |
|  | Yes | 54 | 13 (32) | 28 (68) | 2 (15) | 11 (85) |  |
| Headache intensity | 0 = Very happy, no hurt | 1 | 0 | 0 | 0 | 1 (100) | 0.79 |
|  | 1 = Hurts just a little bit | 15 | 5 (50) | 5 (50) | 1 (20) | 4 (80) |  |
|  | 2 = Hurts a little more | 24 | 6 (32) | 13 (68) | 1 (20) | 4 (80) |  |
|  | 3 = Hurts even more | 9 | 1 (14) | 6 (86) | 0 | 2 (100) |  |
|  | 4 = Hurts a whole lot | 3 | 1 (33) | 2 (67) | 0 | 0 |  |
|  | 5 = Hurts as much as you can imagine | 2 | 0 | 2 (100) | 0 | 0 |  |
| Headache quality | pressure | 4 | 1 (33) | 2 (67) | 0 | 1 (100) | 0.64 |
|  | piercing | 2 | 0 | 1 (100) | 1 (100) | 0 | 0.27 |
|  | throbbing | 44 | 11 (32) | 23 (68) | 1 (10) | 9 (90) | 0.16 |
|  | stabbing | 2 | 0 | 1 (100) | 0 | 1 (100) | 0.66 |
| Headache duration | Several minutes but less than 1 hour | 11 | 3 (32) | 5 (68) | 0 | 3 (100) | 0.53 |
|  | Several hours | 8 | 3 (60) | 2 (40) | 1 (33) | 2 (67) |  |
|  | One day | 5 | 2 (50) | 2 (50) | 0 | 1 (100) |  |
|  | 2-4 days | 21 | 2 (12) | 15 (88) | 1 (25) | 3 (75) |  |
|  | 5-7 days | 8 | 3 (43) | 4 (57) | 0 | 1 (100) |  |
|  | More than 7 days | 1 | 0 | 0 | 0 | 1 (100) |  |
| Headache frequency | Weekly | 3 | 1 (50) | 1 (50) | 1 (100) | 0 | 0.39 |
|  | Monthly | 15 | 3 (25) | 9 (75) | 0 | 3 (100) |  |
|  | Every 2 to 6 months | 26 | 5 (26) | 14 (74) | 1 (12) | 6 (88) |  |
|  | Every 7 to 12 months | 9 | 4 (57) | 3 (43) | 0 | 2 (100) |  |
|  | Less than once per year | 1 | 0 | 0 | 0 | 1 (100) |  |
| Headache with visual disturbances | No | 43 | 11 (33) | 22 (67) | 1 (10) | 9 (90) | 0.72 |
|  | Yes | 11 | 2 (25) | 6 (75) | 1 (33) | 2 (67) |  |

NCC, neurocysticercosis; POC, Point-of-care; TS, *Taenia solium.*

**A5 Table** Epileptic seizures and number of lesions in patients with neurocysticercosis

|  |  | **Any type of NCC (%)** | **Inactive NCC (%)** | **Active NCC** |
| --- | --- | --- | --- | --- |
|  | Epileptic seizures | 7/45 (16) | 3/34 (9) | 4/11 (36) |
|  | *Single lesions* | *1/14 (7)* | *0/13 (0)* | *1/1 (100)* |
|  | *Two to five lesions* | *4/18 (22)* | *3/13 (23)* | *1/5(20)* |
|  | *More than five lesions* | *2/13 (15)* | *0/8 (0)* | *2/5(40)* |
|  | No epileptic seizures | 38/45 (84) | 31/34 (91) | 7/11 (64) |

NCC, neurocysticercosis

**A6 Table** Strobe Checklist

|  | Item No | Recommendation | Page  No |
| --- | --- | --- | --- |
| **Title and abstract** | 1 | (*a*) Indicate the study’s design with a commonly used term in the title or the abstract | 1 |
|  |  | (*b*) Provide in the abstract an informative and balanced summary of what was done and what was found | 2 |
| Introduction | | | |
| Background/rationale | 2 | Explain the scientific background and rationale for the investigation being reported | 3 |
| Objectives | 3 | State specific objectives, including any prespecified hypotheses | 3 |
| Methods | | | |
| Study design | 4 | Present key elements of study design early in the paper | 4 |
| Setting | 5 | Describe the setting, locations, and relevant dates, including periods of recruitment, exposure, follow-up, and data collection | 4 |
| Participants | 6 | *Cross-sectional study*—Give the eligibility criteria, and the sources and methods of selection of participants | 4 |
| Variables | 7 | Clearly define all outcomes, exposures, predictors, potential confounders, and effect modifiers. Give diagnostic criteria, if applicable | 4&5 |
| Data sources/ measurement | 8* | For each variable of interest, give sources of data and details of methods of assessment (measurement). Describe comparability of assessment methods if there is more than one group | NA |
| Bias | 9 | Describe any efforts to address potential sources of bias | 5 |
| Study size | 10 | Explain how the study size was arrived at | 4 |
| Quantitative variables | 11 | Explain how quantitative variables were handled in the analyses. If applicable, describe which groupings were chosen and why | 5 |
| Statistical methods | 12 | (*a*) Describe all statistical methods, including those used to control for confounding | 5 |
|  |  | (*b*) Describe any methods used to examine subgroups and interactions | 5 |
|  |  | (*c*) Explain how missing data were addressed | NA |
|  |  | *Cross-sectional study*—If applicable, describe analytical methods taking account of sampling strategy | 5 |
|  |  | (*e*) Describe any sensitivity analyses | NA |

Continued on next page

| Results | | | |
| --- | --- | --- | --- |
| Participants | 13 | (a) Report numbers of individuals at each stage of study—e.g. numbers potentially eligible, examined for eligibility, confirmed eligible, included in the study, completing follow-up, and analysed | 6 |
|  |  | (b) Give reasons for non-participation at each stage | 7 |
|  |  | (c) Consider use of a flow diagram | Fig 1 |
| Descriptive data | 14 | (a) Give characteristics of study participants (e.g. demographic, clinical, social) and information on exposures and potential confounders | 6,7 |
|  |  | (b) Indicate number of participants with missing data for each variable of interest | Fig 1 |
| Outcome data | 15 | *Cross-sectional study—*Report numbers of outcome events or summary measures | *6-10* |
| Main results | 16 | (*a*) Give unadjusted estimates and, if applicable, confounder-adjusted estimates and their precision (e.g., 95% confidence interval). Make clear which confounders were adjusted for and why they were included | 6-10 |
|  |  | (*b*) Report category boundaries when continuous variables were categorized | NA |
|  |  | (*c*) If relevant, consider translating estimates of relative risk into absolute risk for a meaningful time period | NA |
| Other analyses | 17 | Report other analyses done—e.g. analyses of subgroups and interactions, and sensitivity analyses | NA |
| Discussion | | | |
| Key results | 18 | Summarise key results with reference to study objectives | 11 |
| Limitations | 19 | Discuss limitations of the study, taking into account sources of potential bias or imprecision. Discuss both direction and magnitude of any potential bias | 12 |
| Interpretation | 20 | Give a cautious overall interpretation of results considering objectives, limitations, multiplicity of analyses, results from similar studies, and other relevant evidence | 13 |
| Generalisability | 21 | Discuss the generalisability (external validity) of the study results | 12 |
| Other information | | | |
| Funding | 22 | Give the source of funding and the role of the funders for the present study and, if applicable, for the original study on which the present article is based | 14 |
